# Grandparenting left-behind children in Javanese Migrant-sending villages: Trigenerational care circuits and the negotiation of care

**DOI:** 10.1016/j.geoforum.2023.103767

**Published:** 2023-07

**Authors:** Bittiandra Chand Somaiah, Brenda S.A. Yeoh

**Affiliations:** a Asia Research Institute and Yale-NUS Singapore, National University of Singapore, Singapore; b Asia Research Institute, National University of Singapore, Singapore

**Keywords:** Transnational Families, Grandparenting, Migration, Care, Labour, Divorce

## Abstract

Parental labour migration requires recalibrations of care arrangements within the left-behind family. Existing studies of left-behind families, however, have largely concentrated on parental rather than grandparental caregiving of grandchildren. We argue that grandparents are pivotal to care work and changing family formations within migrant-sending villages. Grandparents provide supplementary care, substitutive care and even reconstitutive care, depending on the migration and marital status of the parents. The paper emphasizes the often unilateral care-contracts between grandparents and migrant parents, drawing on material primarily from the qualitative interviews of grandparent carers of left-behind children, and the grandchildren themselves. By considering a variety of family contexts in flux as a result of parental migration (mother, father or both parents) and marital dissolution amidst migration, we examine family situations holistically by taking into account the different modes of care provided by grandparents (occasionally in tandem with aunts) within changing care contexts.

## 1 Introduction

As transnational labour migration out of East Java, Indonesia, becomes an increasingly common livelihood strategy for rural households, many children are growing up in the absence of one or both migrant parents. As parents are often considered the naturalized carers of children, their absence requires the reconceptualization of intergenerational care in transnational family formations, particularly from the perspectives of those left-behind. In this paper, we seek to understand the role of grandparent caregiving of left-behind children through the concept of ‘intergenerationality’ (Vanderbeck, 2007). Intergenerationality refers to ‘the connections between different age groups or generations and the contingency they have for each other’s social, political, economic and spatial lives’ (Hopkins et al., 2011:314). By drawing on the concept to understand care arrangements when one or both parents are away and/or when a divorce takes place in migrant households, we work towards sharpening the focus on the gendered ‘triadic grandparent-adult child-grandchild relationship’ (Timonen and Arber, 2012: 2), which has often been overshadowed by moralized concerns that centre parent–child relationships. Despite growing evidence that grandparents are involved in emotionally and materially nourishing grandparent-grandchild relations, ‘extra-familial relations such as those with grandparents remain substantially under-researched’ (Tarrant, 2010:192). We thus reinforce calls to ‘situat(e) grandparents’ in social science research, particularly in contexts of increased migration and divorce (Tarrant, 2010:190-191).

We propose the idea of the ‘tri-generationality of caregiving circuits’ in non-Western, labour migration contexts to include multiple generations within the ‘care triangle’ comprising the child, the migrant parent, and the left-behind carer (Graham et al., 2012:797). This enables a better appreciation of how multiple actors of various ages care for one another within transnational households. The term ‘trigenerational care circuit’ not only encompasses transnational family and kin relations, but also emphasizes the inter-connectedness of carework and the caring copresence of multiple left-behind family members. In our fieldwork, we encountered many forms of grandparent care, even if the grandparent was not the main carer. From a broader mixed-methods study of left-behind caregiving within source communities of transnational labour in Southeast Asia, we zoom in on six select households with adolescent left-behind children cared for by grandparents to illustrate the range of grandparental care we observed in the larger study.

How grandparents perform carework in the context of parental migration is significant but under-researched, particularly when migration trajectories are complicated by parental divorce. This paper is thus interested in the caregiving work performed by left-behind elderly in enabling transnational family formations. Caregiving across a skipped generation of absent parents cast a unique light in understanding the conditions of care among those left-behind. Specifically, the research is propelled by three questions. First, how do grandparents supplement care on an everyday basis for left-behind children in transnational families? Second, how do grandparents attempt at substituting care for a grandchild in the long-term absence of one or both parents due to migration? Third, in cases where parental migration is complicated by divorce, how do grandparents reconstitute the familiarity, constancy and ‘normalcy’ of the transnational family?

Before attending to these questions, we first turn to the extant literature on transnational families and intergenerational care in Southeast Asian contexts to consider notions of filial piety, family harmony (*rukun*), and Islamic moral obligations that influence how care circuits are formed in the context of East Java. Next, we look at the literature on gender and grandparenting within gendered care contracts. This is followed by an overview of our research methodology and interview participants. We then offer our analysis of different forms of grandparent care as part of trigenerational care circuits. Our conclusion reemphasizes our arguments and contributions to the transnational family, care geographies, and gerontology literature on gendered and generational left-behind carework, particularly within rural out-migration contexts.

## 2 Grandparents and Intergenerational Care in a Migratory Context

Carework scholars including feminist geographers have noted that ‘increasingly grandparents are playing significant caring roles in contemporary families and are responding to care demands, created by diverse societies that are being shaped in distinctive ways by global trends’ (Tarrant, 2012:182). Intergenerational relations and the left-behind elderly in the context of labour migration however currently remains under-researched despite evidence which ‘suggest that the costs outweigh the benefits as the left-behind elderly are often saddled with looking after their grandchildren’ (Lam et al., 2013:9). Considering this gap in the literature, this paper follows the lead of Schröder-Butterfill (2004:498) to ‘add to the growing evidence for the contributions older people in Asia make to their families’. Poeze et al. too lament that ‘(t)he dynamics and practices involved in creating care arrangements in extended care networks and maintaining relationships between migrant parents and caregivers has been given scant attention in transnational family literature’ (2017:112). This omission is unwarranted, considering that ‘(i)ntergenerational ties and linked lives play an important role in mobility choices’ (Das et al., 2017:1) and more so in the context of Southeast Asia where extra-familial support is a key feature of migrant households. Additionally, it has been acknowledged that ‘(w)hen the young migrate, the elderly are left-behind in need of care’ (ESCWA 2011:4), yet these very elderly, particularly the ‘old old’, are sometimes encumbered with caregiving responsibilities for left-behind grandchildren, thus creating a stretched care situation.

The caregiving experiences of ‘other’ caregivers, particularly grandparent carers, in Southeast Asia have attracted relatively less attention as the focus on how migrants and their left-behind spouses perform ‘mothering’ and ‘fathering’ roles has taken centre-stage (Dewi, 2011, Parreñas, 2001). Often, grandparents occupy the periphery as background carers in relation to migrant mothers whose caregiving dilemmas are often prioritized (Silvey, 2006), or left-behind children, as recipients of care (Beazley et al., 2017). This is beginning to change as seen in emerging scholarship which acknowledges grandparents as carers of left-behind children within care circulations and custodial contexts (Hoang et al., 2015; Teerawichitchainan and Knodel, 2021, Ting and Ho, 2021). Other recent work includes Acedera and Yeoh (2021:182) who drew on the mediated spaces of mobile phones to highlight the role of ‘proximate non-parental carers who help to reconstitute the family amidst growing concerns about adverse consequences of migration-induced care deficits for left-behind children’. Ingersoll-Dayton et al. (2018) explore grandparents’ provision of care in skipped-generation households in Thailand while Peng and Wong (2016) attend to the carework of left-behind Filipino grandmothers. Lawreniuk and Parsons (2017) specifically employ ‘elder translocality’ as a lens to study the active and supportive role of grandparental agency in enabling and facilitating the transnational migration of parents.

In this nascent scholarship, it is usually assumed that the caregivers who step in when mothers migrate are other women, often grandmothers and aunts ‘who replace migrant women in household maintenance, child care and elder care’ (Khoo et al., 2017:339). Precisely because this assumption stems from the view that care is women’s work and is consistent with observed gendered practices of care substitution in Southeast Asian societies, it leaves the gendered dimensions of left-behind grandmothering under-explored while casting a veil of invisibility over left-behind grandfathering. Grandparent caregiving is often equated with grandmother caregiving, and studies on grandparenthood, such Attias-Donfut and Segalen’s (2002) work, tend to underplay the significance of gender in analysing the social construction of grandparenthood. Commenting on the literature on grandparenting in general, Tarrant (2016: 970; see also Harper, 2005) points to the lack of theorizing on grandfathers’ emplaced practices of care, resulting in a ‘limited understanding of the caring spaces in which grandfather care and intergenerational relationships take place’.

While grandparenting has sometimes been portrayed in terms of self-sacrificial acts for the family (Baker et al., 2012; Herlofson and Hagestad, 2012), we refrain from romanticizing the realities of left-behind grandparenting. Rather, in our East Javanese case study, we consider the importance of contextual factors in understanding the nuances of evolving carework that is performed rarely under circumstances of grandparents’ own choosing. This is consistent with Noveria’s (2013) study in West Java that revealed that even if left-behind children lived with their fathers in the wake of their mothers’ migration, the care of young children was mostly taken over by grandmothers. Her study found that ‘grandparents' work to care for the children was unfortunately taken for granted, especially since the majority of these grandparents were not asked about their willingness to accept such a chore’ (Noveria, 2013:10). The caregiving burden ‘simply fell in their laps’ (ibid). As part of the ‘intergenerational contract’ (Croll, 2006) in Indonesia, the grandparental role is rarely monetized or conceptualized as part of a family economic strategy to optimise income gains (Schröder-Butterfill, 2004:516). Rather, grandparental carework when parents migrate is performed out of altruism, service, and/or default absorption of left-behind grandchildren into the care fold. As Schröder-Butterfill observes in the Indonesian context, ‘(o)lder parents do not perceive the option to refuse’ (2004:514). While in many cultural contexts, reciprocal parent-grandparent relations is generally realized through remittances (Agree et al., 2002), it should be noted that in the East Javanese context, monetary remittances are not always distributed equally between left-behind grandparent and left-behind grandchild. Often, a larger portion is often allocated for the needs of children and ‘older parents do not…usually benefit materially from the arrangement’ (Schröder-Butterfill, 2004:514). Instead, grandparents are integral to achieving material migration gains for nuclear families in the next generation, sometimes at the cost of their own well-being. As Schröder-Butterfill (2004: 522) argues, ‘(c)ontrary to the widespread perception that population ageing creates burdens on families, most older people are not dependent’ and instead often provide help during family crisis. In this sense, financially and physically, Javanese grandparents may have more to lose in negotiating intergenerational contracts.

Grandparents’ roles in the family must also need be understood within the economically individualistic culture in Java, where traditionally ‘property is owned individually, family members rarely co-operate economically, and not even all spouses pool incomes’ (Schröder-Butterfill, 2004:514). As observed by scholars of inter-generational family support in rural Indonesia (Keasberry, 2001, Schröder-Butterfill, 2004, Noveria, 2015), the three-generation family under one roof is not a traditional norm, unlike in rural China, where grandparental caregiving ‘not only reflects the strong tie between parents and adult children historically, but also indicates a significant cultural emphasis on collective family interests over individual interests’ (Chen et al., 2011:575). Instead, as Schröder-Butterfill (2004:523) elaborates:

In Java, unlike many other parts of Southeast Asia, there is no preference for extended family households, and where co-residence occurs it is more often a response to vulnerability in the younger, not the older, generation.

Another important contextual factor that needs to be considered in recent decades is the growing significance of *female overseas* migration in rural Java in response to the global marketplace for care and domestic work and the consequences for household formation. While circular migration (*merantau*) is conventionally a masculinist rite-of-passage into adulthood (before marriage), since the 1990s, this practice has become feminized with the Indonesian government hard-selling of low-wage labour migration to meet the global demand for care and domestic work as a rural development strategy (Lindquist 2009). Indonesia now relies heavily on remittances, placed globally as one of the top ten remittance receiving nations (World Bank, 2018:5; World Population Review, 2022). Consistent with the preference for nuclear households, migration is often a remittance-earning strategy to amass money for matrimonial homes. Women’s overseas migration has also focused attention on marital dissolution and the unexpected complications of divorce during migration. Serial overseas migration has been observed to contribute to marital disharmony and divorce, fuelling public discourse around the gendered risks of migration (Nasution, 2019; Somaiah et al., 2020). Conversely, the scholarship on divorce (Nisa, 2011, Platt, 2017) in Indonesia has not given much attention to the role of grandparent caregiving. Here we contribute to the developing literature on kin-work within migrant families experiencing divorce by foregrounding the crucial roles Indonesian grandparents play in filling the caregiving void resulting from parental migration and/or divorce-in-migration.

In light of the above, we address three gaps in the literature. First, we note that ‘it has been far more common to study attitudes towards upward intergenerational support (from adult children to parents, in terms of filial responsibility) than the reverse … from grandparents to grandchildren’ (Herlofson and Hagestad, 2012: 38). Attention to ‘skipped generation’ households have focused on dyadic-generation politics of care, rather than the triadic-generation (Burnette et al., 2013, Uhlenberg and Cheuk, 2010). Even Croll’s optimistic work on the intergenerational contract in contemporary Asian families looks only at the pact as ‘renegotiated and reinterpreted by *both* generations’ (2006: 473) (our emphasis), celebrating ‘a robust and reciprocated cycle of care’ (Croll 2006: 473). In this paper, we highlight trigenerational circuits of care by emphasizing the relationality of care across gender, geographies and three generations. Second, in view of the literature void with respect to grandfathering in the Southeast Asian context, we address this lacuna through shifting some attention to grandfather caregiving. Finally, we address a third gap in the literature by including left-behind grandchildren’s voices, given that ‘of the three generations, (young) grandchildren have to date been accorded least influence in theoretical frameworks’ (Timonen and Arber, 2012: 10) within modern understandings of grandparenting under globalization.

## 3 Research Context and Methods

Overseas labour migration in Indonesia has been actively promoted by the government, principally in its rural areas, as a solution to labour surpluses and to reap the gains of netting foreign currency. For the past four decades, compounded by the 1997 Asian Financial Crisis, migrants from Indonesia have sought contract-based, low-skilled wage opportunities in Southeast and East Asia, and the Middle East, with Malaysia, Taiwan and Hong Kong being the top 3 destinations from 2018 to 2020 (BNP2TKI 2020:13). More than 75% of Indonesia’s 9 million migrants are in low-skilled employment (World Bank, 2017:iii). Most of these are lowly-educated women in the informal sector (UN Stats 2019:5).

This paper draws upon research material from the second wave of the multi-sited, mixed-methods Child Health and Migrant Parents in Southeast Asia (CHAMPSEA) project, conducted in 2016 (quantitative surveys) and in 2017 (qualitative interviews) in rural village field-sites of Ponorogo and Tulungagung (East Java). (While we had planned for fieldwork updates in 2020–21, this was not possible with the advent of the pandemic.) The project looks at the changing care arrangements within households impacted by labour migration. We studied family practices among both transnational and resident households affected by a context of high outmigration. The selected villages consistently possess higher than national average ranks of outmigration (BNP2TKI 2020:29). Our quantitative data of 481 households with adolescents aged 11 to 14 years old revealed that grandparents were the third largest group of carers after mothers and fathers (Table 1). Our survey also found that grandparents were identified as the main carer for households with adolescents in 19 out of 51 divorced households. Grandparent carers were thus very much sought in divorced households in our study, accounting for 37.3 per cent of all primary carers in divorced households. Notably, most of these 19 divorced households had at least one parent who was an internal and/or international migrant. Divorce in the transnational family thus became a significant factor in changing care arrangements that warranted special analysis.

**Table 1:** Primary carers in participating households with child aged 11–14.

**Primary Carer**	**All Households**		**Divorced Households**	
	**No.**	**%**	**No.**	**%**
Mother	361	75.1	20	39.2
Father	71	14.8	5	9.8
Grandparent	36	7.5	19	37.3
Others	13	2.7	7	13.7
Total	481	100	51	100

The main analysis of this paper draws from qualitative, paired interviews with selected households. Household migration status, the gender of the indexed child and well-being outcomes were taken into consideration when selecting a sample of 25 households with a child (aged 11 to 14) for paired qualitative interviews out of the 481 households who were quantitatively surveyed for the project. Children of this age range would still be dependent on caregivers (in comparison to older teens and young adults) yet would be able to express themselves during a conversational style interview context (in comparison to younger children). In each selected household, two separate life-story interviews with the children and their primary carers or adults identified as responsible for the household were conducted. Confidentiality was maintained by having the interviews, conducted by the authors with the assistance of local fieldwork and research assistants, out of earshot of each other. Interviews were conducted in Bahasa Indonesia and in some instances Javanese (a language some elderly participants were more comfortable in). Interviews lasted around an hour on average, and were audio recorded after consent was sought. Each interview was subsequently transcribed, and then translated into English. Pseudonyms are used to protect the privacy of all research participants. Ages of participants are in parenthesis when they are first introduced. While paired interviews from the full sample of 25 households (50 interviews with child and caregiver dyads) inform the broader findings of our paper, here we have chosen to focus specifically on six select households, drawing on 12 interviews with both the children and their carers. These case study households are emblematic of the range of care arrangements we witnessed among our broader group of participants. We provide an analysis of the different modalities of care that grandparents provide and the resultant care relations constructed around left-behind children.

## 4 Supplementary Care

We begin with grandparents’ provision of ‘supplementary care’ in cases where there are already other primary sources of care, including left-behind or return-migrant parents. Grandparent care is unique (Pratt 2009:15) and often actively sought by the grandchildren. Here, grandparents play the role of complementary caregivers, supplementing already obtainable care, so as to guard against care leaks within the transnational family and kin group. In supplementing available parental care, grandparental emotional work and material support cohere with culturally normative nuclear household formations with extended kin help, evident too within non-migrant households interviewed where the primacy of the parent–child care relationships is maintained while grandparents ‘help out’. In the case of transnational families, however, the examples below blur certain normative care norms and expected boundaries, with some children seeming to prefer certain kinds of close grandparental care and proximity over being looked after by their parents.

Gita (60s) helped care for her son’s child, Jenia (14), who was left-behind at the age of two when her father migrated for work. This was a difficult period for the family as the long-distance relationship between Jenia’s father and mother was strained by infidelity, jealousy, and fights. Not only did Gita help Jenia’s left-behind mother with caring for Jenia, she stood by Jenia and her mother, defending them against gossip and malice when the latter became the victim of a rumoured affair circulating within the community. According to Gita, Jenia’s paternal grandfather, now deceased, was also supportive by doing “the man’s job” at home such as fixing lights. When Jenia’s migrant father returned, Gita continued to furnish both physical and emotional care whenever needed. Jenia’s emotional connection with her grandmother is unvarying, so much so that “if she (Jenia) feels angry, she would go to her grandmother” rather than her parents. Gita continues to provide care for Jenia if she is unwell as her mother needs to look after her much younger sibling. While Gita and Jenia do not live in the same household, their homes are right next to each other. Supplementary grandparental care was an important resource during the difficult times of migration and continues to endure even though migration has ceased.

Pertiwi (70s), Anton’s maternal grandmother, became his main carer when Anton’s (12) mother left to work as a domestic helper in Qatar, while his father was (and continues to be) a construction worker in Kalimantan. Anton’s lasting affective relationship with his maternal grandparents is reflected by the fact that even after his migrant mother, Titin (37), returned home after two years and, with her remittances, built a new house in the same village, Anton elected to continue to reside with his grandparents. “He’s been comfortable with his grandma”, Titin reasoned. When Titin called home to enquire about Anton’s health, well-being, and performance at school, much of the communication depended on Pertiwi as the go-between: if Anton had any problems, he would tell Pertiwi who would then share them with Titin. When she first returned, Titin disclosed that initially, Anton was not very close to her – “It took quite some time, … yes indeed… maybe because of the fact that he was left behind… *Alhamdulillah* (Praise be to Allah) we’re close now. *Alhamdulillah*, it was like that”. It is with gratitude and relief that Titin shared that mother and son have managed to forge closer bonds after her return. Still, Anton’s close relationship with his grandmother persisted. Anton revealed that it is Pertiwi who provides him with daily pocket money while he saves the money that his parents give him monthly. He also helps Pertiwi in her work collecting firewood, drying rice and coconut, and feeding the livestock. Anton recalled that it is Pertiwi, together with his parents, who takes him to the doctor when he falls ill. For Jenia and Anton, grandparental care lingers beyond migration; it does not supplant but supplements parental care.

## 5 Substitutive Care

Beyond providing supplementary care, grandparental care can also intensify in some migrant households to the extent that care is substitutive in nature, replacing the care of a migrant parent in both substance and emotions. Grandmothers may step in to replace migrant mothers, upholding the conventional woman-carer model. Rukiyah (55), Wiwid’s (14) paternal grandmother, raised Wiwid in her own house since she was born, having been left-behind by her mother when she was only a month old. Wiwid’s parents were both migrants. While her mother is still a migrant domestic worker in Hong Kong, her father has returned but is living separately from Wiwid. Rukiyah has thus become Wiwid’s substitute carer, a replacement mother in the absence of her birth mother. Rukiyah observed that ‘she’s (Wiwid) not close to her mum’, and indeed Wiwid herself confirmed that in the family, she is closest to her grandmother. Even when her mother comes home on leave, she continues to stay and sleep at her grandparents’. While Rukiyah received money from Wiwid’s mother for school fees, pocket money and daily expenses, Rukiyah is willing to provide financial support if needed, saying, “If she doesn’t have it, I’ll use my money… I give her whatever amount I can give”.

We observed a similar instance of substitutive grandmaternal care in the following household. When Rusli’s (14) mother left to undertake domestic work in Hong Kong, he was ten months old. It was Kusuma (67, widowed), Rusli’s maternal grandmother, who cushioned the heartbreak:

She gave birth…breastfed [Rusli]… And then she left…She first wanted to leave when he was 1-month old, but she couldn’t bear the thought of leaving him. She cried when she was leaving. I took the two children out to play when she left.

Rusli’s mother told Kusuma that she wanted Kusuma to care for her children. The initial care-contract was thus unilaterally proposed and accepted, in a context where grandparents are “morally expected” and assumed to be “ready” to perform carework for grandchildren. At that time, Kusuma complied, taking it for granted that she had to assume caregiving duties when Rusli’s mother left. Rusli’s mother visits every two years, but each time for only a couple of weeks.

Kusuma lives with Rusli, his brother, and their left-behind father, a duck farmer and rice-field labourer who is illiterate. She takes care of Rusli, cooks for him, and looks after him when he is unwell while also coping with the housework. She struggles to discipline Rusli, who she describes as “*kasar*” (rough). She was the one who explained his mother’s migration to him, just before he entered school. When asked, Rusli professed he feels closest to her in the household, and that “both grandmother and father” raised him.

Kusuma observed that, “when [Rusli’s] mother asked him to sleep in the same bedroom [during her home visits] … he didn’t want to [be with her] … he didn’t want to be close with his mother.” His mother had bought him a phone, but Rusli who has not seen her “since he was a baby… didn’t really care for her”. While he uses the wi-fi his mother has installed in the house, Rusli has yet to save her Hong Kong phone number in his phone. Kusuma is concerned about the gradual commodification of this migrant parent-left-behind child relationship which also extends tri-generationally to encompass the relationship between migrant parent and left-behind grandmother. While she too is a recipient of gifts such as a necklace from her daughter, she would rather have her daughter home. The remittances sent home by Rusli’s mother have translated into raised living standards in the form of a new house, and a 700-square-meter rice field and ploughing machine to augment her husband’s work. For Kusuma, however, the provision of things appears insufficient to truly nurture trigenerational ties and intimacy.

While different kinds of care, embodied and long-distance, are enacted by both the grandmother and mother respectively, it is embodied care which seems qualitatively privileged by both Rusli and Kusuma. Of Rusli’s mother, Kusuma rued “There’s not much love. She only sends money”. She has grown bitter over the years in her views of her absent daughter’s sense of maternal responsibility:

What responsibility? She’s [Rusli’s mother] not even here! Once, she told me she wanted to return home, but she couldn’t (laughing)… She said her employer’s house was about to be sold [implying that a maid is no longer needed after the move]. [She told me,] ‘Mother, what do I do if my employer sells the house?’ [I told her,] ‘Come back home.’ [She said,] ‘Oh, Mother, don’t say that.’

Although Kusuma summed up her own role in the family in minimalist terms as “I cook the rice…I cook the side dishes”, she is in fact substituting the care of a mother. Her equating of carework with feeding work (Somaiah, 2022) was a common theme for all the grandmothers interviewed. Yet her carework encompassed so much more, including the emotional labour that comes with caring for teenage grandsons – dismay that one of her other grandsons has dropped out of school to be a farmer, and hope that Rusli will persevere and do well. While there are clearly benefits from remittance money, Kusuma feels increasingly stressed by her grandmothering responsibilities to two rebellious teenage grandsons and yearns for her daughter’s return.

Like Wiwid and Rusli, many left-behind children in the broader sample spoke of receiving grandparental care at some stage of the migrant parent’s absence, particularly during the early childhood years. When sustained over time, there is a reorienting of the primary care relationship towards the grandparent (often the grandmother in line with the feminization of carework), as a result of the long-term absence of the migrant parent (usually the mother rather than father), as well as the abdication of certain kinds of (emotional) care by the left-behind parent (often the father rather than the mother). As with supplementary caregiving, material care (such as remittances, gifts, and wi-fi provision) still seems to flow primarily between the migrant parent and the left-behind parent and/or left-behind child, sometimes bypassing the grandparent carer.

## 6 Reconstitutive Care (Amidst Divorce)

While grandparents play an important role in supplementing or substituting parental care in transnational families where one or both parents are away generating material support, they become pivotal to the ability of the family to successfully reconstitute itself in cases where care re-arrangements resulting from parental migration are further disrupted by marital dissolution. While Brickell makes occasional inferences to grandparental support in focusing on the familial politics around divorce or home ‘unmaking’ (2014:262), as does Locke et al. (2014:281), we emphasize the significant role of grandparental caregiving in transnational home ‘re-making’ (Brickell, 2014: 270) in contexts with left-behind children.

In Indonesia, prolonged international migration has been linked to marital problems and divorce (Hugo, 2005). Migration, as distance apart and time away, is considered a contributor to divorce, and this is further exacerbated by culturally inflected economic factors when women as migrant breadwinners earn substantially more than their spouses and therefore possess higher purchasing power within the marriage. Migration is thus considered to impact the *rukun* (culturally valued social peace and harmony) of the home to the extent that some districts in Indonesia are pushing for migrant women to write letters promising that they will not file for divorce while abroad. While gendered double standards have long existed when it comes to divorce, this is especially accentuated when the divorce is initiated by the migrant mother. Popular discourse, fuelled by the mass media, has been alarmist to a large degree in reporting how local governing bodies and left-behind husbands have been caught unawares by divorce-seeking migrant wives. Vernacular Indonesian newspaper articles often assume that divorce in migration is migrant-wife instigated, with some news items lamenting the bad fortune of husbands whose wives leave them and their children due to affairs abroad with PIL, an acronym for *Pria Idaman Lain* (other dream men). Such discourses inevitably portray husbands/fathers as naively left-behind, in contrast to bad wives/mothers who are depicted as greedy or impatient to earn quick money abroad. The trope of the bad migrant mother is evident in the media, where marital dissolution is seen to lie at the doorstep of migrant women.

Up until five years ago when her parents divorced, Ambar (12) was cared for by her left-behind father, receiving only supplementary care from her paternal grandparents. Ambar’s mother left to work in Taiwan when Ambar was just a toddler. Upon the discovery that her mother was having an extramarital affair in Taiwan, her father strategically migrated to Taiwan for work in an attempt to salvage the marriage. According to Ambar’s current carer, her paternal grandmother, Nining (63), it took Ambar’s father two months to locate her mother in Taiwan but a reconciliation was impossible as he found out that ‘she had already been together with another man’. In the absence of both parents, Ambar was moved to her grandparents’ house and Nining assumed the main carer role for as long as her father had been working in Taiwan prior to and following the divorce. This was also in spite of the fact that her mother has returned to the village for close to two years, having remarried but living in her natal household not far from Ambar. When asked how she felt about being the primary carer for Ambar, Nining shared,

Her father’s away…her mother’s not here anymore…As a grandmother I have to take care of her.

Nining was accepting of her caregiving role, particularly amidst divorce. To her, the role of a grandmother is simply ‘taking care of the children, taking care of the grandchildren’. This matter-of-fact attitude towards active and involved grandparenting echoes Hoang, Yeoh and Wattie’s (2012) observation that grandparents are critical in enabling continued migration, especially in Indonesia where a ‘simple transfer of care’ from parent (in Ambar’s case, her father) to grandparent (usually grandmother), is taken for granted as a natural course of action. A crisis of care did not erupt in Ambar’s household because of Nining’s willingness to fill the caregiving gap when the marriage broke down. By taking over the carer role from Ambar’s father, he could then focus on working in Taiwan to provide financially for Ambar. As Nining noted, ‘Well, now that her father is [working] abroad, there’ll be money if his daughter wants something.’ Ambar herself affirms that her father tops up her phone credits directly from his own phone account whilst in Taiwan. She also shares that importantly ‘her father wants her to continue her studies…until [she] attends university’ and to this end, he has already bought an education savings plan for her using his remittances from migration. Nining’s active role as a grandmother taking care of Ambar is therefore the enabler for her father’ continued migration in Taiwan. Whilst Nining mentions that Ambar’s return-migrant (but remarried) mother fetches her home from school every day on her motorcycle, Nining feels that Ambar and her mother are not emotionally close:

She doesn’t want to live with her mother… [She’s close] to her father. She never cries if her mother goes overseas…When it’s her father…then she’ll definitely cry.

The strained relationship between mother and daughter is also evident in our interview with Ambar when she opted not to respond to any questions relating to her mother (while readily sharing other aspects of her life). With her grandmother, Ambar has a comfortable but not close relationship: she stated that she does “nothing” with her grandmother apart from helping out “sometimes” with household chores like sweeping, but recalled her grandmother’s meticulous care for her when she was hospitalized. In turn, Nining is finding caring for Ambar as she grows into her adolescent years “easy up until now (laughs)”. She spoke with mixed feelings of Ambar’s growing independence, for instance, Ambar rides her motorcycle (gifted by her father) to her afternoon extra-curricular tuition classes which is ‘nearby’ even though she is still young (and not of legal age to ride), largely ‘because there’s nobody who can bring her’ (neither grandparent can ride due to their old age and health). Ambar’s behaviour echoes other studies on left-behind children who strive for independence, if only to reduce the burden on their left-behind carers. Nining appreciates Ambar’s resilience yet confides, “If she goes out, I’ll worry about her”. A previous study on grandparental confluences of worry around left-behind caregiving in Thailand (Ingersoll-Dayton et al. 2020) illustrate the myriad of anxieties which burden such grandparents. Emotional labour exacted by stressful undercurrents is part and parcel of reconstituting the family in the wake of divorce, particularly where there are care gaps which cannot be easily bridged.

The extension and reconstitution of care relations is also apparent in the next case of divorce-in-migration. Farmer Heriyanto (87), is the self-identified maternal grandfather-carer for Beny (14) who was left-behind at the age of five when his mother left for Saudi Arabia to work as a domestic helper while his father worked in Malaysia. Beny’s mother was in Saudi Arabia for seven continuous years, and did not return even once. When she did finally return three years ago, it was to proceed with a divorce triggered by her husband’s infidelity (Heriyanto admitted of his son-in-law, “He couldn’t resist… when his wife went [to Saudi Arabia] and he was working in Malaysia, he found another wife”). She then left again this time for Malaysia to continue working as a domestic worker for the past two years, post-divorce.

Heriyanto lives with Beny in the house owned by Beny’s mother and acts as a guardian of the remittances sent for Beny. Heriyanto’s daughter’s earnings abroad are meant only for Beny – she remits 500 000 Rupiah (S$50) monthly for Beny’s school fees and other needs. Beny’s father also sends the same amount for Beny’s upkeep – and has continued to do so post-divorce – but to another relative from whom Heriyanto has to collect the money each month. Beny receives a small sum as pocket money from Heriyanto and goes to his grandfather when he wants to buy things. Beny often asked Heriyanto for more money to fill up gas for his motorcycle, for example, to which Heriyanto would accede, reasoning that “he is still young after all”. While financial support tends to flow from migrant parents to the left-behind child with the grandparent acting as steward to manage (but not benefit from) the money, emotional care to meet the child’s needs lies in the hands of the proximate non-parent carer. Heriyanto is content with this arrangement, saying that all he wants is to uphold *rukun* (the Javanese term for culturally valued social harmony): “I don’t want anything. I just want to take care of those kids”. Meanwhile, the grandchild-grandparent care relationship is sustained by a certain reciprocity. Appreciative of his grandfather’s care, Beny enjoys keeping his grandfather company such as watching television programmes together, and helping his grandfather feed his 20 catfish or sweep the house. In turn, Heriyanto affirmed that caring for Beny is not difficult as he is not naughty and listens to his advice.

Heriyanto’s wife was Beny’s principal caregiver until she passed away three years ago. Heriyanto inherited the grandparenting carework from her while also enlisting the help of one of his daughters, Wening, on her return from Brunei where she was working. Other relatives too pitched in. By lending Beny a laptop to complete homework, Wening plays a crucial role as the disciplinarian in the absence of Beny’s *bapak* (father who is culturally endorsed to take charge of the discipline of children) and in the presence of an ageing grandfather figure. Living a stone’s throw away from Beny, Wening has taken on board the appeal of Beny’s mother to “take care of him just like you take care of your own children”. With respect to Beny’s relationship to his mother, Wening confided, “his mother loves Beny actually. But because Beny was left when he was young, he’s shy with his mum when he is older. He’s closer to me than his mum…He’s afraid of me. I always remind him of time to study, to pray”. As an aunt-caregiver she takes on both the caregiving and disciplinary aspects of the parenting dyad, while Heriyanto takes on the paternal role of providing (remitted) money to Beny while also providing companionship and a daily presence. In this household, the tri-generational care arrangement extends beyond the migrant parent–carer–left-behind child ‘care triangle’ (Graham et al. 2012) to take the form of a ‘web of care’ comprising multiple caregivers in relation to the child linked together through circuits of communicative care. This multifaceted modality of care adheres to the gendered and feminized construction of carework – including the key roles of left-behind aunts and grandmothers – within rural patriarchal contexts as highlighted by previous work (Chen, 2022), but also acknowledges that carework reconstituted under conditions of duress can and do cross the gender divide to involve grandfathers and other male relatives.

The trigenerational care circuit re-emplaces care-chains and intergenerational care within the (extended) family, in this case including grandparents who provide care. The notion of ‘trigenerational care circuits’ re-familiarizes care within the (extended) household rather than focus on markets of care worker supply (Ogawa et al. 2018) and *trans*-border movement of care labour (Ortiga et al., 2021). We home in on the family relations of transnational families who rely heavily on left-behind grandparents. These grandparents provide rock-solid support, even reconstituting the family in unanticipated ways. The trigenerational care circuit represents the relationalities between various members of the transnational household including non-migrant, non-parent carers, particularly kin-carers within or near households of left-behind children. This conceptualisation helps us shift some attention to grandparent caregiving, destabilizing the prevailing view centring the migrant in terms of purchasing power in supplying care, and the left-behind child as the idealized recipient of the earnings gleaned from migration for purposes such as education.

Since her inclusion in Beny’s trigenerational care circuit, Wening has played a central role in decision-making relating to Beny’s well-being, although always in symbolic deference to Heriyanto’s authority. Recently, concerned that Beny may be led astray by the wrong crowd, she has insisted that he sleeps over at her house so that she can keep a watchful eye over him. Stemming from these concerns, she is also planning on sending him to a *pesantren* (Islamic boarding school) soon and has taken active steps to secure the agreement of Beny’s mother:

I told him to go to Islamic boarding school. I don’t think I’m able to take care of him every day…I’m scared of his problematic friends… I’m worried about him…I decided after he graduated from MTs (Madrasah Tsanawiyah or Junior High Islamic School), I’ll send him to Islamic boarding school.

In the context of parental absence complicated by divorce-in-migration, both grandfather and aunt play complementary care roles in reconstituting family for the left-behind child. The trigenerational care circuit is arranged to sustain the left-behind child until he is ready to move to an institutionalized care context. Divorce-in-migration thus reconstitutes care by including more non-parental caregivers in the remaking of family.

Through their reliable if taken-for-granted carework, grandparent carers often play a pivotal role in reconstituting ‘broken’ families of migration. Migration changes the contours and ‘normative structures of familial and intergenerational relationships’ (Alipio et al., 2015: 255). As shown in the cases of Beny (maternal migration) and Ambar (paternal continued migration), fostering out or adopting-out (Beazley and Ball, 2022) children to reliable next of kin is an important enabler (Lawreniuk and Parsons, 2017) of parental migration. Beyond its enabling function, reconstitutive care by grandparents leads us to question the seemingly persistent ideology of motherhood and mother-care as irreplaceable, a cultural narrative which Francisco-Menchavez argues maternal migration reinforces rather than destabilizes (2019:95; see also Parreñas, 2005, Chan, 2017, Yeoh et al., 2020).

In the face of the wear and tear that families in labour migration confront, we see left-behind grandparents take caregiving in their stride, as do left-behind children for whom life carries on with fewer interruptions thanks to the constancy of proximate caregivers such as grandparents and aunts. While Zhao et al. (2017: 675) note in the context of left-behind children in China that the ‘availability of support…plays an essential role in child well-being’, it was ambiguous if grandparents were perceived by children to have been pillars of social support during divorce, partly due to the fact that grandparents were the constant primary carers (regardless whether parents were at home or away for work) in contexts even before divorce occurred. In our study, grandparents play a particularly vital role in sustaining and reconstituting family care relations in cases of divorce-in-migration through their day-to-day presence, embodied caregiving, foodwork and emotion work. As other scholars such as Platt (2017) have shown, (re)configurations of the institution of marriage and the family have historically been expansive, inclusive, and relatively more liberal in rural Indonesia. Grandparental caregiving for left-behind children from divorced households is just one incarnation of multiple family forms and systems of care in East Java. Unlike supplementary and substitutive forms of care, both emotional and material care relationships are reconfigured to centre the grandparent-grandchild relationship in reconstitutive modes of care. Here, there seems to be an attenuation of children’s emotional and material bonds to at least one, if not both parents, in light of family dissolution. As care deficits risked by migration and divorce develop, care reparations are ably met by grandparents.

## 7 Parental Migration and Changing Trigenerational Care Contracts

Here, while our findings tend to corroborate previous results from a gendered care perspective, we have extended our focus to a wider range of elder care-enabling and elder caregiving. While often cast as a ‘soft’ demographic in ageist depictions within the migration literature as being passively left-behind, grandparents are providing a variety of care in migration contexts: first, as supplementary broader kin care-buffers for children affected by parental migration; second, in terms of substituting mostly physically absent mothers; and third, in reconstituting the family in circumstances of pre- and post-divorce. Alongside grandparents, relatives, particularly aunts, were also adapting their caregiving to the changing care politics that emerge around the needs of growing grandchildren, nieces, and nephews.

Croll lauds that presently ‘the new exchange of care is openly acknowledged to be more balanced and symmetric, with both generations, simultaneously or in shortened cycles, giving and receiving care,’ offering a ‘new framework for reciprocity’ (2006: 484). This was not necessarily the case for our older participants. Grandparents’ emotional, mental, and physical challenges often went unnoticed, unheeded, and unexpressed. One grandmother carer in a divorced household, Suparmin (61), confided, “I was sick when her mom departed there…After she migrated, I was sick immediately…Yes. It was like that too when she and her ex-husband migrated for the first time”. In this instance, migration itself was the trigger for this grandmother’s high blood pressure, stroke, and numerous ailments. We thus challenge the notion that bridging work is occurring across generations in the migratory context of rural Java. Rather, the cleavages between and across the three generations are more apparent for these rural left-behind poor in East Java. While the intergenerational contract can be seen to be malleable, it is also morphing for migrant families, and sometimes the care emanating from the grandparent generation is the one which is most conveniently taken for granted.

Each type of grandparental care mentioned in this paper involves different parent-grandparent-grandchild gendered and generational politics of care, depending on which parent has migrated, and whether marriages are still intact or dissolved. It is arguable if the transactional notion of the ‘intergenerational contract’ has much valence in the everyday realities of rural grandparents. Rather, grandparents tend to see themselves as largely self-reliant individuals, asking for money only when required (for example for medicines). Grandparents’ heavy emotional investment in their (grand)children’s well-being ‘means that they are not passive ‘recipients’ of the transnational care arrangement, but play active parts in its formation and implementation’ (Kassaye 2015:50). Grandparent carers in our study are enabling parents, particularly mothers, to widen their breadwinning capacities through labour migration. This is at the paradoxical expense of grandmothers in particular, assuming traditional feminized carework so as to facilitate their daughters’ enhancement of earning power and in turn social status. They enable continued migration to occur, and also enable children to develop well by forming reciprocal emotional bonds with their wards through everyday, embodied carework. While there are some challenges expressed around discipline, particularly among teenage boys, and technology issues (Acedera and Yeoh, 2021), most of the grandparents interviewed feel a sense of fulfilment caring for their grandchildren, and speak about them with pride. Spending time (even if only watching television programmes together) with left-behind children is also a form of embodied care. We thus add to incipient literature on the ‘intersections of physical and social space in intergenerational contract’ (Tarrant, 2010:192).

As illustrated through the case studies, reconstituting family in the aftermath of divorce under migration is an important service rendered by grandparents for left-behind children of dissolved marriages broken in migration. Supplementary, substitutive and reconstitutive care from grandparents, particularly grandmothers, can be considered as part of a repertoire of ‘adaptive strategies that the transnational family pursues in order to cope with the reproductive vacuum left behind by the migrant [parent]’ (Hoang et al., 2012:733). We have argued firstly that all the three types of care charted in our paper are crucial to the well-being and survival of the transnational family, particularly under the stress of divorce. Even in non-divorce households, the supplementary care and availability of a caring grandparent is a comfort to left-behind children. The seeking of both emotional and physical shelter and solace midst migrating parents is a boon for the transnational family – whether it means enjoying a grandmother’s meal, or the warmth of her bed. Secondly, particularly in the case of migrant mothers, we have shown how often a grandmother plays the role of surrogate mother by substituting maternal with grandmaternal caregiving work including cooking, feeding and emotional labour. The substitution carework can be so convincing and effective in allowing children to form secure attachments and bonds with the grandmother that it effectively replaces the role of the mother. Perhaps as an unintentional survival strategy, the mother is framed as emotionally insignificant and relegated to being a remittance-sender. Thirdly, we have argued that grandparental caring for left-behind children in the context of divorce is critical in holding the family together in its new form even if this kind of care is feminized, at the expense of their health, and even if remittances are mostly channelled to children’s needs and bank accounts via other kin and the physical, practical gains are minimal for the caregivers.

This imbalance is softened by reciprocal care relations between grandchild and grandparent who through sharing time and space together, form close emotional bonds. These are meshed with trickier parental ‘intergenerational solidarities that are simultaneously transnational’ (Binnie and Klesse, 2013:593). Grandparental physical and embodied caregiving complements parental financial support for children’s future educational pathways, and more immediate day-to-day expenses. The embodied presence of a grandparent can counterbalance the physical absence of a migrant parent. Counter-factually, if grandparents were not accepting these grand-parenting caregiving roles, parents might not be able to continue migration to the desired benefit of helping children achieve their educational aspirations. Among our broader group of participants, feelings of abandonment, neglect, loneliness and helplessness among left-behind children were common as were walls of silence or amnesia when broaching the topic of parental migration. In some cases, unhappiness and emotional phone-calls to parents to return home had indeed sparked some migrant parents’ return.

Substitutive care and reconstitutive care provided by grandparents accentuates their roles vis-à-vis left-behind children more than supplementary care, which tends to be embedded into the natural mis-en-scene of care networks in translocal village life. Of note too in dealing with potential snares in care safety nets is the presence of aunt-carers who complement, uphold and support the care-giving and authority of the caregiver grandparent, particularly when stamina fails. Murphy too notes how care gaps can occur when grandparenting left-behind children, for example in relation to not being able to manage grandchildren’s school demands due to being illiterate (2022:187). Nonetheless salient, pragmatic caregiving from grandparents ensure the continuity of family for children of migration, particularly in the aftershock of divorce. At the risk of (self-)exploitation, but with the potential for emotional gains such as comfort and company, sympathy for the left-behind grandparent caregivers must be conjoined to respect for their significant roles in maintaining the trigenerational integrity of the (divorced) household in migration and its everyday intimacies surrounding ever-evolving politics of care – even if the feminized script persists to the peril left-behind grandmothers. As Suparmin, a grandmother carer who suffers ill-health from a stroke asserted, “Hopefully my care for them will pass from generation to generation”. This paper has sought to conceptualize more inclusive, expansive, and encompassing roles the elderly play in the abiding, caring ‘reproductive mobilities’ (Sheller, 2018) of our time. Altruism, ambivalence, and acceptance are interwoven in the kinds of supplementary, substitutive and reconstitutive care given by grandparents (most of whom are grandmothers) towards their left-behind grandchildren.

## 8 Conclusion

Our paper has contributed to three main research gaps we identified. Firstly, this paper makes not just an empirical, but conceptual contribution to studies of grandparental caregiving in the wake of parental migration, and within Southeast Asia specifically. Secondly, while Tarrant employs gender analytically in researching grandparenting to understand how grandfathering is temporally and spatially constructed via carescapes in England (2013), here we look at Indonesian left-behind grandmothering with one instance of grandfathering. This reflects current de jure gendered, feminized grandparenting norms but more studies are needed to better understand grandfather caregiving in diverse contexts. Our paper examines grandparental intergenerational caring relations, unfair care contracts, and the gendered costs of this to contribute to gaps in understanding these delicate care negotiations. Thirdly, we include where possible, left-behind adolescent grandchildren’s voices to situate their perspectives to being grandparented. Though their voices need amplification since that is often for them that parental migration is embarked upon, it is beyond the scope of this current paper. For now, to better make sense of the realities of left-behind grandparenting from migrant-sending villages in Java, and the three forms of grandparental care that emerged, we offer the concept idea of trigenerational care circuits as vital within negotiations of transnational family carework. Additionally, left-behind grandparenting carework can be viewed as an intervention to counter disciplining discourses around maternal labour migrants as ‘bad mothers’ common in mass-mediated moral panics around the changing family under migration in this instance in Indonesia, but also in other high migrant-sending nations like the Philippines.

Gender has emerged as a prime factor of analysis in studies of migration (Kofman and Raghuram, 2022: 281). We have incorporated within our gendered analysis of migration and care, an additional gendered analysis of left-behind grandparents’ caregiving of children. We add to growing work around the role of grandmothers in caregiving within contexts of migration. While Tyldum (2015) studies grandmothers who became paid carers to fund younger Ukrainian women’s migration to Italy under contexts of divorce, in our study, grandmothers absorb the aftershocks of migration and divorce through their unpaid care. While we intended to study grandparenting within participating grandparent-carer households, what emerged were mostly grand*mother*-carer households, overrepresented too in divorced households. Gender thus became the lens to understand the topic of left-behind grandmothering and experiences of these carers who perform invaluable gendered care. Acknowledging that grandparental care for children is gendered (Harman et al., 2022), we add to the growing care work literature on ‘gendered moral rationalities in later life’ (Hamilton and Suthersan, 2021) and accompanying cultural expectations (Abdul-Malak, 2017) by shifting attention to the source communities of carework migrants in rural Java.

## Declaration of Competing Interest

The authors declare that they have no known competing financial interests or personal relationships that could have appeared to influence the work reported in this paper.

## Data Availability

The data that has been used is confidential.
